# Changes of Gene Expression in *Euglena gracilis* Obtained During the 29^th^ DLR Parabolic Flight Campaign

**DOI:** 10.1038/s41598-019-50611-4

**Published:** 2019-10-03

**Authors:** Julia Krüger, Peter Richter, Julia Stoltze, Sebastian M. Strauch, Marcus Krüger, Viktor Daiker, Binod Prasad, Sophia Sonnewald, Stephen Reid, Michael Lebert

**Affiliations:** 1 Cell Biology Division: Gravitational Biology Group, Department of Biology, Friedrich-Alexander University Erlangen-Nürnberg, Staudtstraße 5, 91058 Erlangen, Germany; 2 Postgraduate Program in Health and Environment, University of Joinville Region, Rua Paulo Malschitzki, 10 - Zona Industrial Norte, Joinville, SC CEP 89219-710 Brazil; 3 Clinic for Plastic, Aesthetic and Hand Surgery, Otto von Guericke University Magdeburg, Leipziger Straße 44, 39120 Magdeburg, Germany; 4 Biochemistry Division, Department of Biology, Friedrich-Alexander University Erlangen-Nürnberg, Staudtstraße 5, 91058 Erlangen, Germany

**Keywords:** Cell biology, Transcriptomics

## Abstract

Parabolic flight maneuvers of Novespace’s Airbus A310 ZERO-G produce subsequent phases of hypergravity (about 20 s), microgravity (about 22 s) and another 20 s hypergravity on experiments located in the experiment area of the aircraft. The 29^th^ DLR parabolic flight campaign consisted of four consecutive flight days with thirty-one parabolas each day. *Euglena gracilis* cells were fixed with TRIzol during different acceleration conditions at the first and the last parabola of each flight. Samples were collected and analyzed with microarrays for one-color gene expression analysis. The data indicate significant changes in gene expression in *E*. *gracilis* within short time. Hierarchical clustering shows that changes induced by the different accelerations yield reproducible effects at independent flight days. Transcription differed between the first and last parabolas indicating adaptation effects in the course of the flight. Different gene groups were found to be affected in different phases of the parabolic flight, among others, genes involved in signal transduction, calcium signaling, transport mechanisms, metabolic pathways, and stress-response as well as membrane and cytoskeletal proteins. In addition, transcripts of other areas, e.g., DNA and protein modification, were altered. The study contributes to the understanding of short-term effects of microgravity and different accelerations on cells at a molecular level.

## Introduction

*Euglena gracilis* is a unicellular freshwater flagellate, which is distantly related to kinetoplastids, a group of parasitic protozoans, such as *Trypanosoma* or *Leishmania*^1,2^. It shows distinct movement behavior with respect to external stimuli, such as light, oxygen, and acceleration^3–5^ and has been a model organism for biological clocks^6,7^, photosynthesis^8,9^, and movement physiology^10,11^. In the absence of light, cells show negative gravitactic behavior, adjusting the swimming direction against the vector of acceleration. Both gravitaxis and the light-directed phototaxis in *E*. *gracilis* are based on active physiological mechanisms^10,12–18^. However, a large part of the underlying signal transduction chain remains to be elucidated. So far, a distinct calmodulin, as well as a particular protein kinase A, were found to be involved in graviorientation^12,13^. In addition, a flagellar protein has been implicated in the process^19^. Furthermore, the contribution of mechanosensitive ion channels, changing membrane potentials and calcium seems very likely^16,17^.

Previous parabolic flight campaigns studied the movement and physiological parameters of *E*. *gracilis*: in the 45^th^ ESA parabolic flight campaign, a change in the beating pattern of the *E*. *gracilis’* flagellum during the transition from hypergravity (hyper-*g*) to microgravity (μ*g*) and vice-versa was observed^18^. Earlier, in the 29^th^ ESA parabolic flight campaign^16^, changes in membrane potential, and the cytosolic calcium concentration were measured during different accelerations. Furthermore, movement analysis revealed a fast and precise adaptation of the swimming behavior depending on the prevailing acceleration^17^. Negative gravitaxis was more pronounced during hyper-*g*, while µ*g* led to a random movement as compared to a 1 *g* control sample. While these studies confirmed the physiological nature of the gravitactic orientation, no studies on differential gene expression in *E*. *gracilis* had been performed in the course of parabolic flights. This was in large part due to the lack of complete genomic and transcriptomic datasets of *E*. *gracilis*. However, very recent advances in the genome and transcriptome sequencing of *E*. *gracilis* allowed the analysis of global gene expression pattern induced by different stimuli^2,20–22^. In this study, we used this knowledge to investigate changes in *E*. *gracilis* gene expression in parabolic flights using a large-scale microarray analysis system. We show that short time accelerations or microgravity is sufficient to cause significant changes in gene expression in *E*. *gracilis*.

## Results

### Hierarchical clustering

**Figure 1:**
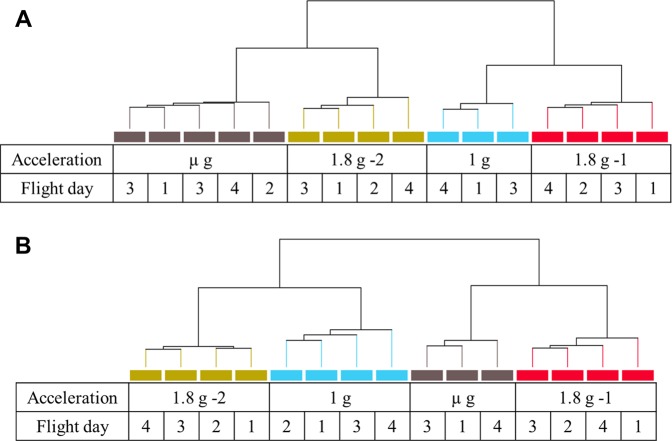
Hierarchical clustering of gene expression data of parabolic flight samples from parabola 1 (**A**) and 31 (**B**). Independent samples cluster together based on the subjected acceleration irrespective of the flight day for both parabolas. (A) For parabola 1, µ*g* (grey bars) and 1.8 *g*-2 (green) show close resemblance as well as 1 *g* (blue) and 1.8 *g*-1 (red). (**B**) In parabola 31, clusters appear for 1.8 *g*-2 (green) and 1 *g* (blue) as well as µ*g* (grey) and 1.8 *g*-1 (red). Hierarchical clustering was performed based on normalized intensity values using Pearson uncentered correlation and Wards linkage rules. At least three independent samples for each acceleration from different flight days were employed for hierarchical clustering.

To correct for any adaptational effects, individual accelerations in the first and last parabolas were analyzed independently. After removal of all outliers, at least 3 samples for each acceleration were employed for hierarchical clustering. Cell cultures subjected to the same acceleration within a parabola showed the highest degree of similarity independent of the flight day (Fig. 1). For parabola 1, samples of the µ*g* phase showed a close resemblance to the second hyper-*g* phase (1.8 *g*-2), while 1 *g* and the first hyper-*g* phase (1.8 *g*-1) exhibited closer relations. In parabola 31, these similarity clusters changed since 1.8 *g*-2 and 1 *g*, as well as µ*g* and 1.8 *g*-1, appear to relate to each other.

### Comparison of gene expression changes between parabola 1 and 31

**Figure 2:**
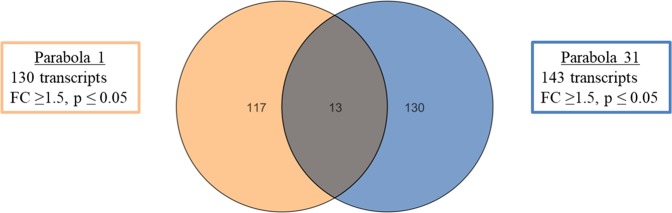
Overall change of gene expressions between all significantly (p < 0.05) differentially expressed genes (FC > 1.5) in the first (#1, orange circle) and last parabolas (#31, blue circle), respectively. Differentially expressed genes of all phases (1 *g*, µ*g* and 1.8 *g*) durin*g* parabolas were pooled. The intersection between the two circles show the number of genes that were affected in both the parabolas 1 and 31.

Comparison of gene expression changes between parabola 1 and 31 showed very distinct differences. Overall, 260 genes were found to be significantly regulated during the first and last parabola by a fold change of ≥1.5 and a p-value ≤ 0.05. About 130 genes met these criteria during parabola 1, while 143 transcripts were regulated in parabola 31 (Fig. 2). Only 13 transcripts were found to be regulated during both parabolas (Table 1). However, these regulations occurred in different phases within the parabolas (1.8 *g*-1, µ*g*, 1.8 *g*-2) and showed contrary fold changes in parabola 1 and 31. Four of the transcripts were in good accordance, when their protein sequences were compared in the BLAST database to sequences of other organisms, like carboxyl-terminal-processing peptidase chloroplastic-like isoform X2 (#7821, E-value 2.4E-60), molybdopterin adenylyltransferase (#14765, E-value 3.1E-85), GPI-anchor surface protein (#627, E- value 1.8E-109), and mitogen-activated kinase kinase kinase NPK1 (#450, E- value 4.3E-50). An adenylate guanylate cyclase domain-containing protein (#1113, E-value 9.2E-07), as well as an acyl-binding domain-containing protein 5-like (#30616, E-value 1.6E-09), showed low similarity. Seven of the regulated transcripts showed no similarities to other known proteins or only weak similarities to hypothetical proteins. Therefore, no annotation was possible.

**Table 1:** Significantly co-regulated transcripts during parabola 1 and 31.

Transcript number #	Description	Parabola 1			Parabola 31		
		1.8 *g*-1	µ*g*	1.8 *g*-2	1.8 *g*-1	µ*g*	1.8 *g*-2
7821	Peptidase chloroplastic-like isoform X2*			1.99 ↑			−1.52 ↓
14765	Molybdopterin adenylyltransferase	−1.51 ↓				−1.75 ↓	
627	GPI-anchored surface	−1.98 ↓	1.78 ↑				−1.95 ↓
450	Mitogen-activated kinase kinase kinase NPK1			1.72 ↑			
1113	Adenylate guanylate cyclase domain-containing	−1.56 ↓					1.70 ↑
30616	Acyl-binding domain-containing 5-like		1.62 ↑				1.72 ↑
34765	Hypothetical protein	1.56 ↑					−1.76 ↓
32090	Hypothetical protein			−1,54 ↓	1,56 ↑		
20366	NA	−1,58 ↓					1,52 ↑
41001	NA		−1.74 ↓			−1.75 ↓	
29283	NA		−1.53 ↓			1.62 ↑	−1.63 ↓
16124	NA		1.58 ↑				1.67 ↑
15640	NA			1.52 ↑↓		1.60 ↑	

The description of co-regulated transcripts (#) is found with the Blast2GO search. The fold change of up- or down-regulated (↑ or ↓, respectively) transcripts at different phases (1.8 *g*-1, µ*g*, 1.8 *g*-2) during the parabola 1 and 31 is presented as well.

NA, not available; *carboxyl-terminal-processing peptidase chloroplastic-like isoform X2.

The overall distribution of significantly regulated genes in parabola 1 and 31 is shown in Table 2. The changes were relatively small but distinct. In the course of the first parabola, no changes beyond 2-fold were detected and the number of regulated transcripts was similar for the different accelerations. Lower numbers of genes showed regulation more than 2-fold during various accelerations in the last parabola. These included BMP family ABC transporter substrate-binding protein (EG_35058), Slh1p interacting factor (EG_15691) and ubiquitin-conjugating enzyme E2 Z-like (EG_3282). For further analysis, fold changes above 1.5 were closely investigated. During the first hyper-*g* phase (1.8 *g*-1), 51 transcripts showed significant changes in expression, while in the subsequent µ*g* and second hyper-*g* (1.8 *g*-2) phases, 42 and 46 transcripts were altered, respectively. In the last parabola, the number of regulated genes increased with the duration of the parabola, starting from 29 transcripts in the first hyper-*g* phase to 51 during the µ*g* phase to 81 transcripts in the second hyper-*g* (1.8 *g*-2) phase.

**Table 2:** Overall distribution of significantly regulated (p < 0.05) genes for different fold changes during different acceleration phases in the parabola 1 and 31.

Fold Changes (FC)	Parabola 1			Parabola 31		
	1.8 *g*-1	µ*g*	1.8 *g*-2	1.8 *g*-1	µ*g*	1.8 *g*-2
FC > 1.1	206	630	248	212	302	723
FC > 1.5	51	42	46	29	51	81
FC > 2.0	0	0	0	1	2	3

Each parabola consists of a first hyper-*g* phase (1.8 *g*-1) of 20 s, a µ*g* phase of 22 s and a second hyper-*g* phase of 20 s (1.8 *g*-2).

### Gene expression changes in parabola 1

**Figure 3:**
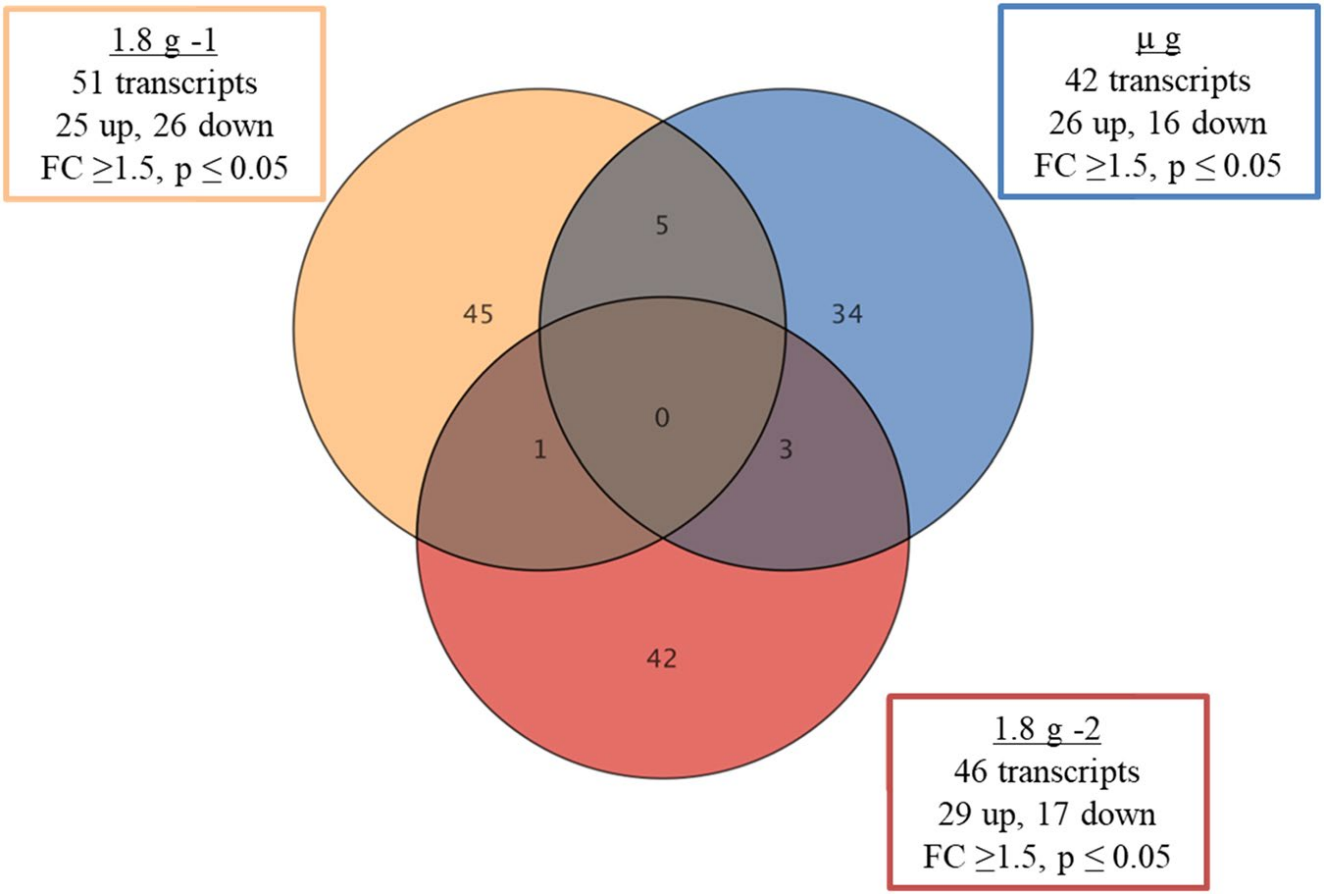
Gene expression changes during different acceleration phases of the first parabola. Most of the gene expression changes are specific for a particular acceleration phase. Each circle represents an acceleration phase (orange = first 1.8 *g*, blue = µ*g*, and red = second 1.8 *g* phase). Intersections between circles show the number of genes that changed in more than one acceleration. A fold change cut-off of 1.5-fold and a p-value of 0.05 were applied using moderated t-test.

In total, 130 transcripts were significantly altered under various accelerations in parabola 1 (Fig. 3), indicating that changes are acceleration- as well as time-dependent. In this regard, only very few genes were regulated in more than one phase. For instance, among all significantly regulated transcripts, only a single one was regulated in both hyper-*g* phases. Furthermore, during µ*g* and the second hyper-*g* (1.8 *g*-2) phase, up-regulations of transcripts were more pronounced than down-regulations. During µ*g* phase, 26 transcripts were up-regulated and 16 down-regulated and in the second hyper-*g* phase, 29 transcripts showed up-regulation, while only 17 exhibited down-regulation.

Table 3 summarizes the annotation of domains or functions of all altered transcripts in parabola 1. For some transcripts, Blast2GO found no similarities to any of the known proteins, which were marked as not applicable (NA). For others, it was not possible to assign a function by means of Gene Ontology (GO) annotation or manual inspection (marked as no GO) due to poor annotation. Therefore, sequences were checked manually and revised if necessary. The conserved domains or gene ontologies were determined for about 50% of all transcripts. During the first parabola, most altered transcripts were involved in signal transduction processes and transport mechanisms. Furthermore, nuclear processes including DNA binding, nuclear localization, and DNA replication, recombination and repair as well as transcription were also regulated throughout the entire parabola. Interestingly, photosynthesis-associated transcripts, as well as a blue-light using FAD (BLUF)-domain proteins, were also affected although samples were fixed in the dark. Gene set enrichment analysis (GSEA) found only one enriched GO for DNA metabolic processes (GO:0006259) during the microgravity phase in parabola 1.

**Table 3:** Transcript function and domain annotation of the first parabola.

Function	Parabola 1		
	1.8 *g*-1	µ*g*	1.8 *g*-2
Amino acid biosynthesis	1		
Biosynthesis	1		
BLUF-domain protein	1		
Chemotaxis			1
DNA-binding			1
DNA replication, recombination, repair	3	3	1
Membrane protein	1	3	
Metabolism			2
Nuclease activity			1
Nucleic acid binding	1		
Nuclear localization			1
Peptidase	1		
Photosynthesis	1		1
Protein degradation		1	
Protein folding			1
Protein glycosylation	1		
Protein targeting			1
Posttranslational modification	2		
Signal transduction	7	3	5
Stress protein			1
Transcription		1	3
Translation		1	3
Transport	7	5	1
Vacuolar sorting	1		
No GO	5	6	3
NA	18	19	20
**Total**	**51**	**42**	**46**

Annotation was performed with Blast2GO or manually based on BLAST search. Genes with no or weak similarities as found by Blast2GO annotations were marked as not applicable (NA) and genes with poor annotation and/or no conserved domains were referred to as no Gene Ontology (no GO). The E-value threshold for all annotation was set to 1E-03.

A list of transcripts found to be significantly regulated during the different acceleration phases of parabola 1 as well as their corresponding sequence description, organism and similarity measure (E-value) as determined by Blast2GO or manual BLAST search are shown in Table S1. The affected signal transduction elements included different protein kinases, adenylate and guanylate cyclases, and a single phosphodiesterase. Furthermore, possible prokaryotic sensor histidine kinases (*E*. *gracilis* transcript numbers EG_15442 and EG_836) were up-regulated during both hyper-*g* phases. Affected transport mechanisms included elements involved in the intracellular transport as well as the transport of macromolecules and ions across membranes (Table S1). Interestingly, a BLUF domain protein was down-regulated (EG_31756), showing similarities to *E*. *gracilis* PACα subunit, involved in the light-directed movement called phototaxis. Furthermore, a transcript similar to *E*. *gracilis* chloroplast light-harvesting complex II precursor Lhcbm3 (EG_41739) was also down-regulated. A very high similarity of the transcript EG_627 was found to a GPI-anchor surface protein, which was down-regulated in the first hyper-*g* and up-regulated in the subsequent µ*g* period. During the second hyper-*g*, an up-regulation of a possible fructose-1,6-bisphosphate aldolase (EG_43521) was found.

### Gene expression changes in parabola 31

**Figure 4:**
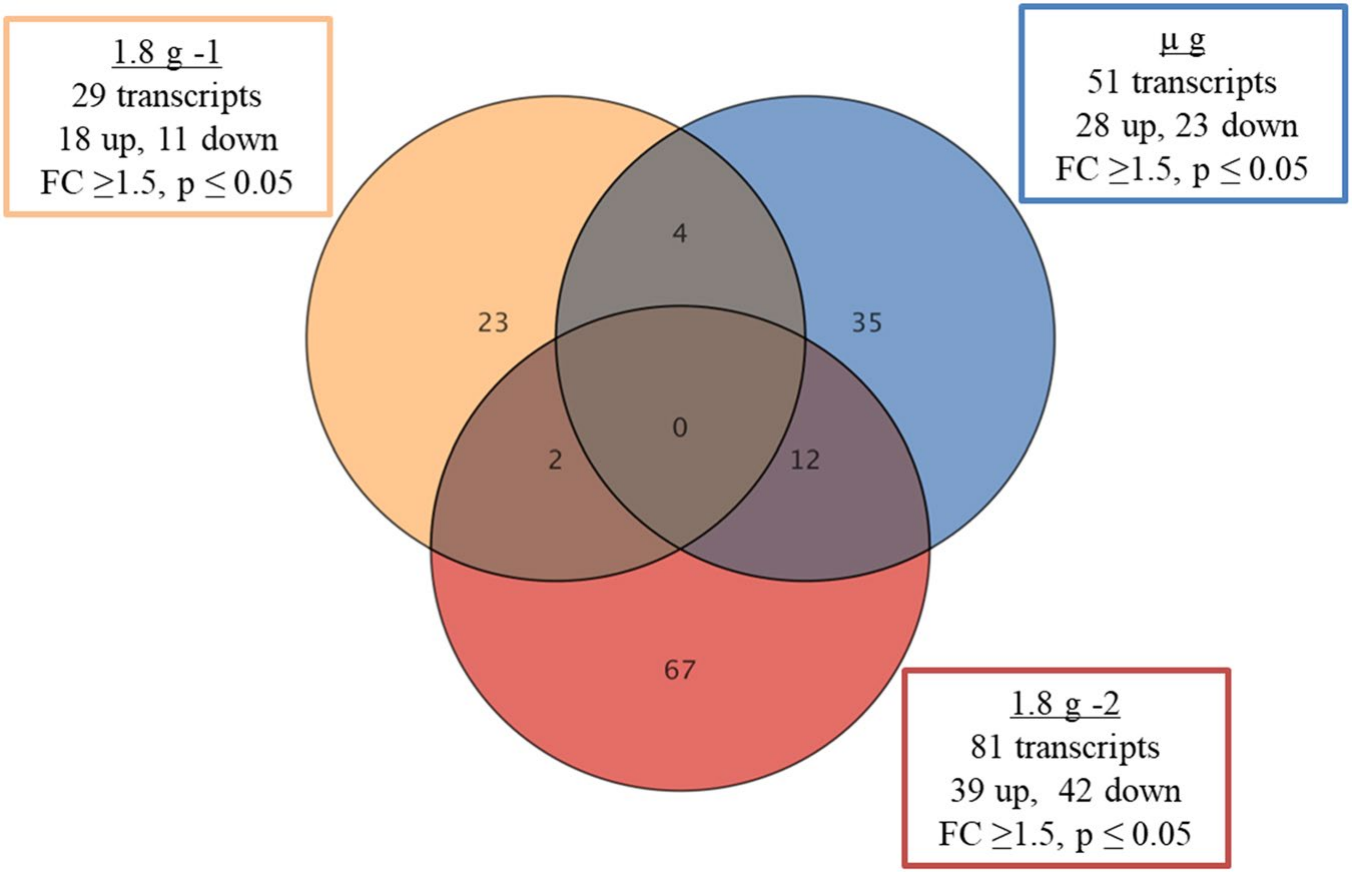
Gene expression changes during different acceleration phases of parabola 31. Most of the gene expression changes are specific for a particular acceleration phase. Each circle represents an acceleration phase (orange = first 1.8 *g*, blue = µ*g*, and red = second 1.8 *g* phase). Intersections between circles show the number of genes that changed in more than one acceleration. A fold change cut-off of 1.5-fold and a p-value of 0.05 were applied using moderated t-test.

In parabola 31, 143 transcripts significantly regulated. The number of altered transcripts increased with the duration of the parabola. In the initial hyper-*g* phase, 29 transcripts were regulated, while the µ*g* and the last hyper-*g* phase affected 51 and 81 transcripts, respectively (Fig. 4). Furthermore, up- and down-regulations were uniformly distributed between different phases.

Table 4 summarizes the annotation of transcripts for parabola 31. Protein domains or gene ontologies were found only for about 50% of the transcripts. However, none of those showed similarities to transcripts with annotations in *E*. *gracilis*. Thus, it was necessary to rely on similarities to protein descriptions found in other organisms (E-value). In addition, no significantly regulated Gene Ontology terms were found in the GSEA analysis and therefore, the analysis focused on single transcript validation (Table S2). Most pronounced effects were found for transcripts related to signal transduction elements, protein modification, membrane proteins, transport mechanisms, and calcium-binding proteins. Among the signal transduction elements were different kinases, phosphatases, cyclases, and a PAS sensor histidine kinase. Large subsets of transcripts were found to be involved in protein ubiquitination processes acting as ligases or hydrolases but were only regulated during both hyper-*g* phases. The analysis of transport mechanisms revealed that mainly active export mechanisms were affected as most transport proteins showed to have an ATP-binding cassette. Further, a possible ammonia transporter was found. Membrane proteins included a GPI-anchor surface protein (EG_627), with very high similarity scores in BLAST search. It was down-regulated in the second hyper-*g* phase. While in the 1.8 *g*-1 and µ*g* phases, single stress proteins (EG_14035 and EG_1003) were found, no regulation for such mechanisms was seen during the second hyper-*g* phase. During µ*g* and 1.8 *g*-2 phases, a NADP malic enzyme (EG_8836) functioning in energy production and conversion was up-regulated.

**Table 4:** Transcript functions and domain annotation of parabola 31.

Function	Parabola 31		
	1.8 *g*-1	µ*g*	1.8 *g*-2
Actin binding			1
Amino acid biosynthesis	1		
Amino acid transport and metabolism			1
Biosynthesis		1	1
Calcium-binding protein		2	3
Carbohydrate transport and metabolism		1	
Cell differentiation			1
Chromatin organization			1
Cytoskeleton			1
DNA replication, recombination, repair	1	2	1
Energy production and conversion		1	1
Galactosidase oxidase domain			1
Glycosyltransferase		1	1
Lipid transport and metabolism			2
Membrane protein	2	1	3
Microtubuli binding			1
Motorprotein		1	1
mRNA binding		1	
mRNA splicing		1	
Nucleic acid binding			1
Peptidase			2
Protease activity			1
Protein modification	4		5
RNA binding			3
RNA modification	1		
Secretory protein			1
Secondary metabolite synthesis		1	
Signal transduction	2	8	11
Stress protein	1	1	
Transcription	1	1	1
Translation	1	1	1
Transport	2	2	2
Vesicle trafficking			2
No GO	2	6	6
NA	11	19	25
**Total**	**29**	**51**	**81**

Annotation was performed with Blast2GO or manually based on BLAST search. Genes with no or weak similarities as found by Blast2GO annotations were marked as not applicable (NA) and genes with poor annotation and/or no conserved domains were referred to as no Gene Ontology (no GO). The E-value threshold for all annotation was set to 1E-03.

## Discussion

Earlier experiments performed on parabolic flights with the model organism *E*. *gracilis* have shown that it can undergo a very fast adaptation of its swimming behavior during the course of a parabola, which is coupled to intracellular mechanisms^16–18^. In the recent 29^th^ DLR parabolic flight campaign, this knowledge was broadened by the analysis of gene expression changes. The data obtained revealed that the short-term alterations of acceleration induce significant changes in gene expression in *E*. *gracilis*, in particular of transcripts involved in signal transduction, transport mechanism, metabolic pathways and stress-response. Changes in gene expression within seconds of microgravity obtained with parabolic flights have been reported previously in callus cultures of *Arabidopsis thaliana*^23^ as well as in human cell lines^24,25^.

### Gene expression changes in *E*. *gracilis* correspond to observations in other organisms

The data partially accord with previously obtained gene expression changes on the spacecraft Shenzhou 8, where *E*. *gracilis* cells were fixed after 45 min of exposure to microgravity^26^. Quantitative PCR analysis revealed effects on the genes involved in signal transduction, calcium signaling, stress response, heat shock, and cell cycle proteins. In this study, regulation of different signal transduction elements, such as mitogen-activated kinase kinase kinase NPK1, hybrid sensor histidine kinase/response regulator, serine/threonine-protein kinase, adenylate guanylate cyclase domain-containing protein, cyclin-dependent kinase-like 2, PAS domain-containing sensor histidine kinase, etc., was observed in *E*. *gracilis* during different phases of a parabola. However, these changes cannot be directly linked to a distinct pathway, as the only recently accessible transcriptome data lack comprehensive annotations^21^. Interestingly, some of the transcripts found to be regulated in *E*. *gracilis* showed similarities to specific observations involved in hyper-*g* and μ*g* experiments in other cell types. For example, effects on the primary metabolism were found to be induced by parabolic flights. In callus cultures of *Arabidopsis thaliana*, changes in the phosphorylation pattern of malate dehydrogenase were investigated^23^, which also showed altered transcriptional levels (EG_8836) in *E*. *gracilis* in the present study. Furthermore, a potential fructose-1,6-bisphosphate aldolase 1 (EG_transcript_43521), acting in glycolysis, was up-regulated in the second hyper-*g* phase during parabola 1. Interestingly, higher transcript levels and an increase in the activity of a product produced by this enzyme, dihydroxyacetone phosphate, which is an activator of pyruvate kinase was found during a sounding rocket campaign in *Arabidopsis* cell cultures^27,28^. This could emphasize the assumption of an increase in glycolytic turnover.

Another aspect well studied under altered gravitational conditions is the production of reactive oxygen species (ROS), which have been identified to function as a second messenger in the signal transduction of gravitational changes^23,29–31^. We also found transcripts involved in the production of ROS regulated during parabola 31 (EG_1003; EG_14035) indicated as stress proteins. However, the production of ROS and transcripts involved in the protection against damages induced by free oxygen radicals seems less pronounced and the latter was not found to be significantly regulated. During a space mission with samples fixed after 45 min, the ROS involved transcripts were found to be regulated in a more significant manner^26^.

The up-regulation of an ammonium transporter (EG_1810) during the second hyper-*g* phase is consistent with the data found for *A*. *thaliana* cell cultures during a TEXUS campaign and was associated with a stress-induced up-regulation of protein synthesis^28^. This could be in agreement with the pronounced regulation of elements (e.g. E3 ubiquitin-ligases and ubiquitin carboxyl-terminal hydrolases) of protein modification processes found in parabola 31 as well as transcripts associated with the transcription and translation in the first and last parabola.

Interestingly, transcripts involved in photosynthesis and transcripts containing a BLUF domain, which is associated with light-directed movement in *Euglena*, were regulated in the course of the first parabola. Although cells were kept in dark during the whole experiment, differences in regulation were found between the first sample (1 *g* control) and the following acceleration phases (1.8 *g*-1, µ*g*, 1.8 *g*-2). This could indicate interconnectivity between the impact of changed accelerations and perception of light (^13,19^).

### Different responses between parabola 1 and 31

In general, results from the first parabola vary significantly from transcripts regulated during parabola 31. Only 13 out of 271 transcripts were regulated during both parabolas. In the time between, cells experienced a further 29 parabolas during a 2 h period, which could possibly lead to an adaptation of their physiological state to the encountered acceleration changes, and could therefore explain the different responses. Although in-flight movement analysis was not performed in this campaign, previous parabolic flight campaigns have shown no differences in the swimming behavior during the various parabolas^17^. This would indicate that the adaption is limited to the transcriptional and most likely also to the protein level (unpublished data). Within the parabolas, the varying accelerations (1.8 *g*-1, µ*g*, 1.8 *g*-2) also induced very specific effects with only a few transcripts being regulated under more than one phase. In parabola 1 and 31, no transcripts were regulated in all phases of acceleration. Most pronounced overlaps occur in subsequent phases during either of the parabolas, indicating a time-dependent effect. This could be emphasized by the fact that at least during parabola 31, the number of regulated transcripts increases with the progression of the parabola. Interestingly, most of the co-regulated transcripts showed the opposite direction of regulations in the phases.

### Fast acceleration changes during a parabolic flight are sufficient to generate distinct gene expression changes

The measured gene expression changes occur within a timeframe of 20 s. Even though this seems to be too short for profound alterations, other studies have also found similar effects^23,32–34^. From numbers reported in literature, Thiel *et al*.^35^ concluded that these changes are plausible if the responsible signaling cascades only take seconds to transduce into the nucleus and the chromatin structure allows immediate binding of transcription factors. The mRNA synthesis by RNA polymerases II has been shown to be within the time frame of a single acceleration during a parabola^36–39^. A further relevant step towards a translatable mRNA is the pre-mRNA processing by splicing, which may be the most time-consuming step. However, recent reports about co-transcriptional splicing show that transcriptional speed also regulates splicing and that it can be conducted much faster than expected (reviewed in^40,41^). So far, time constants for splicing vary greatly between different cell types and methods used for measurement, making further investigations necessary, but one of the fastest splicing durations was found in HeLa cells with an average of 30 s^42^. Furthermore, measurements of pre-mRNA levels, as well as process other than the *de novo* synthesis of mRNA, have to be considered when analyzing rapid gene expression changes, as found during a parabolic flight.

As Thiel *et al*.^35^ concluded, a prerequisite for rapid gene expression changes is a fast mechanical transduction from the membrane, where acceleration changes are possibly perceived, into the nucleus. They referred to predictions from the tensegrity model, which describes a force propagation of 30 m s^−1^ along the pre-stressed cytoskeleton and studies linking changes in cell geometry with changes in nuclear and chromatin structure and gene expression changes (reviewed in^43–46^). At an acceleration of 1 *g*, elastic fibers of the cytoskeleton, such as microtubules, are under tensional loading. Sudden transfer to µ*g* unloads the compressed fibers leading to cytoskeletal reorganization and changes in cell geometry^47–49^. The cytoskeleton in *E*. *gracilis* has been shown to be directly related to changes in cell geometry^50^. However, predictions from the tensegrity model for a free-swimming organism like *E*. *gracilis* need to be investigated further. In this study, we found changes in cell surface proteins (EG_10146, EG_627, EG_1870), the cytoskeleton and their interacting proteins (EG_800, EG_14051, EG_22888, EG_23041, EG_12478, EG_38089), as well as DNA-binding (EG_31054), and chromatin organization domains (EG_26357) during various acceleration phases, indicating a possible role of cytoskeletal components in transduction of mechanical forces in *E*. *gracilis*.

The conformity of the results in the independent cell samples taken at independent subsequent flight days presents a strong argument that the data are not just due to the random fluctuations or aberrations during sample preparation and measurement, flight vibrations, changes in light regime, subsequent phases of altered accelerations and/or mechanical stress of cells being transferred into the flight hardware. In addition, the similarity of gene expression changes of independent cell samples at distinct phases of a parabola was much closer than the conformity of gene expression changes of the same sample at different phases of acceleration.

## Conclusion

It is known that *E*. *gracilis* reacts to changes in acceleration by adaptation of its swimming behavior. However, it was so far unknown whether these conditions induce changes at the transcriptional level. This study shows for the first time that gene expression in *E*. *gracilis* is capable of global dynamic alterations induced by parabolic flights. Results indicated significant changes in gene expression within a very small time. Changes in acceleration for ~20 s resulted in up to two-fold up- or down-regulations of particular genes in *Euglena*. In addition, the transcription differed between the first and the last parabolas indicating adaptation effects in the course of the flight. It was also observed that different phases of the parabolic flight affected different gene groups, which corresponds to data found in other organisms such as those involved in signal transduction, calcium signaling, transport mechanisms, metabolic pathways, stress-response, membrane and cytoskeletal proteins, and DNA and protein modification. Taken together, this study contributes to the understanding of gene expression changes under altered accelerations and microgravity and can be used as a reference data set for further on-ground and space flight related studies.

## Material and Methods

### Cells and growth conditions

*E*. *gracilis* KLEBS strain Z (obtained from the SAG, Göttingen) was grown in 150 mL complex medium^51^ in 300 mL Erlenmeyer flasks. A fresh culture was inoculated for every flight day with 3 × 10^6^ cells per 150 mL of medium. Cell number was determined using a Thoma chamber (VWR, Radnor, Pennsylvania, USA). To generate a controlled growth condition, a portable, isolated plastic container was used as a culture chamber. The culture was grown at 20 °C and defined constant light of 25 W/m^2^ was achieved with mixed warm-white and cold-white illumination (Solarox, Stendal, Germany; same mix and intensity as in the cell culture room used for stock cultures in the laboratory). The experiments were performed with eight days old cultures showing pronounced negative gravitaxis.

### Parabolic flight campaign

**Figure 5:**
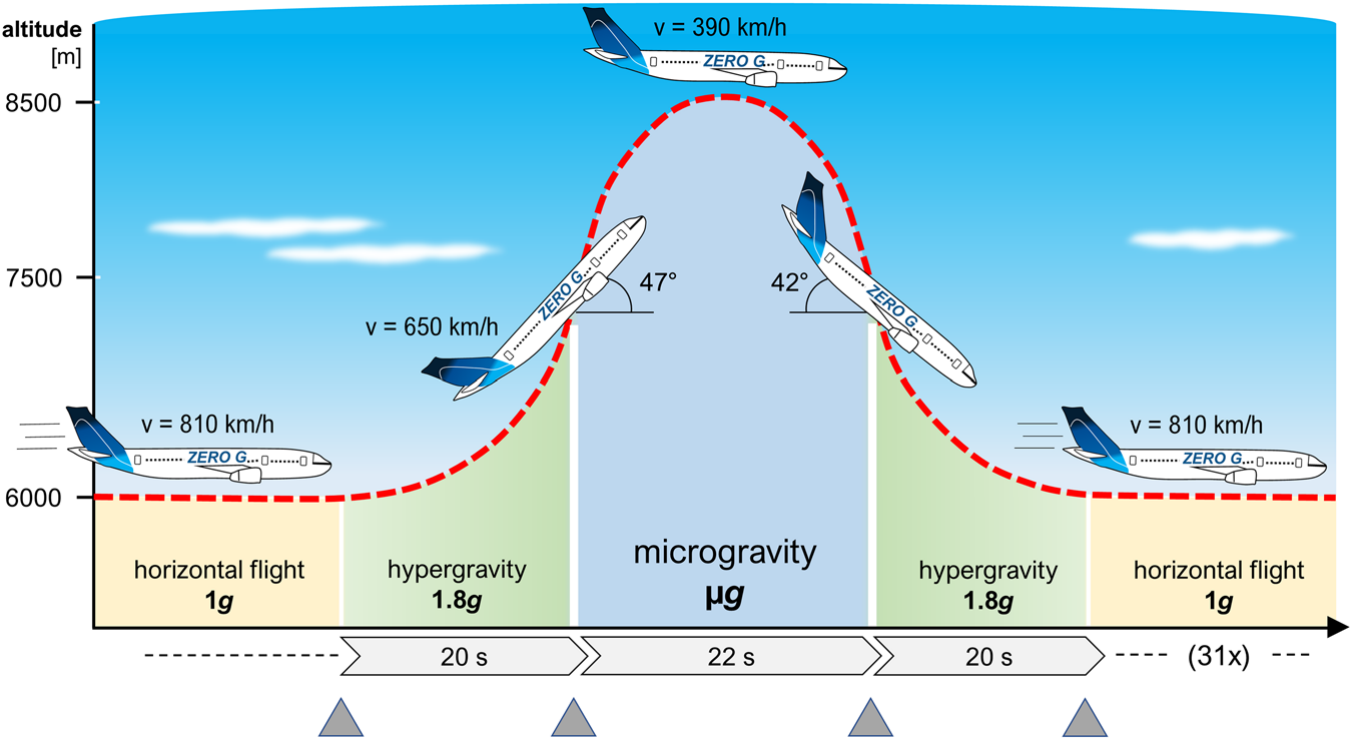
Schematic representation of the acceleration levels of a typical parabola. Grey triangles indicate the sampling events at the end of 1 *g*, at the end of the first hyper-*g* phase of 20 s, at the end of the µ*g* phase of 22 s, and at the end of the second hyper-*g* phase of 20 s. These sampling events were repeated for the 31^st^ parabola as well.

Experiments were conducted in the course of the 29^th^ DLR parabolic flight campaign in September 2016 in Bordeaux, France. A modified Airbus A310 performed flight maneuvers resulting in short periods (22 s) of µ*g*, flanked by two hyper-*g* periods (each about 20 s) before and after the µ*g* phase (Fig. 5). Detailed descriptions about parabolic flight campaigns are provided in Pletser *et al*.^52^. The schedule of the campaign was as follows: Ten days sample preparation as well as validation of the experiments in terms of safety followed by four subsequent flight days with 31 parabolas each day.

### Experimental rack, filling and fixation procedure

For inflight fixation of samples, a syringe-based fixation unit was used, allowing manual release of a fixative into syringes containing *E*. *gracilis* cells. During preflight preparations, 2 mL of cells from the cell culture flask were carefully filled into 20 mL syringes (Henke-Sass Wolf GmbH, Tuttlingen, Germany). Each syringe was sealed with parafilm and connected by serological tubing (Extension line type Heidelberger, B. Braun Melsungen AG, Melsungen Germany) to another 20 mL syringe containing 13 mL TRIzol^®^ (Life Technologies, Karlsbad, USA) as a fixative. The syringe pairs were mounted into a custom-made holder, which provided space for 16 syringe-pairs in total (Fig. S1), allowing fixation of eight independent samples in duplicates. The syringe holder was integrated into the experimental rack, constructed according to the safety requirements. For fixation, TRIzol^®^ was released from one syringe into the counterpart containing cells. The applied pressure destroyed the parafilm membrane. Due to safety requirements, the pistons of the syringes were activated by sliders, which connect the inside of the box mechanically with the outside. The sliders were pushed manually in defined time intervals during the first and the last parabola. The fixation time points for each of the parabolas were chosen as follows: first fixation step: 1 *g* phase ten seconds before onset of hyper-*g*, second step: end of hyper-*g* phase (20 s 1.8 *g*), third step: end of µ*g* phase (20 s of µ*g*) and fourth step: end of second hyper-*g* phase (20 s hyper-*g*) (Fig. S1). Each fixation step was performed in duplicate. All four consecutive flight days were used to collect sample material. Samples were stored at -80 °C and shipped on dry ice from Bordeaux, France to Erlangen, Germany for further analysis.

### RNA isolation

For the isolation of total RNA from cells, 2.6 mL of chloroform (Merck, Darmstadt, Germany) were added to all parabolic flight samples. Samples were shaken vigorously for 15 s and incubated for 3 min at room temperature. Following this, the upper aqueous phase obtained after centrifugation at 14,000 × *g* and 4 °C for 40 min was transferred into a fresh tube and RNA was precipitated by the addition of 2 mL isopropanol (Sigma-Aldrich, St. Louis, USA). Samples were carefully inverted and incubated at room temperature for 10 min, followed by centrifugation at 12,000 × *g* and 4 °C for 30 min. Subsequently, the RNA pellet was washed twice with 75% ethanol (VWR, Radnor, USA), dried at 50 °C and resuspended in 20 µL of nuclease-free water (Thermo Fischer Scientific, Waltham, USA). RNA concentration and quality were checked using the NanoDrop™ lite spectrophotometer (Thermo Fischer Scientific, Waltham, USA) and the Agilent 2100 Bioanalyzer (Agilent Technologies, Santa Clara, USA) according to the Agilent Technologies RNA 6000 Nano Assay protocol.

### cDNA-synthesis and synthesis of fluorescent complementary RNA (cRNA) for microarray analysis

For hybridization on a microarray, fluorescent cRNA was generated using the Low Input Quick Amp Labeling Kit (Agilent Technologies, Santa Clara, USA) according to manufacturer’s instruction. In brief, 100 ng of total RNA was complemented with 2 µL spike-in RNA (One-color RNA Spike-In Kit, Agilent Technologies, Santa Clara, USA), and a T7 primer mix. Primer and template RNA were denaturized for 10 min at 65 °C, followed by 5 min incubation on ice. For reverse transcription, 5x first-strand buffer, 0.1 M DTT, 10 mM dNTP mix, and Affinity Script RNase Block Mix were added to the RNA mix and the reaction was performed at 40 °C for 2 h, followed by heat inactivation of the reverse transcriptase at 70 °C for 15 min. In a subsequent *in-vitro* transcription step, the cDNA was used as a template in order to produce Cy3-labeled cRNA. The cDNA was mixed with T7 polymerase, a transcription master mix, and Cy3-labelled CTP and incubated for 2 h at 40 °C. Resulting cRNA was column purified using the RNeasy Mini Kit (Qiagen, Hilden, Germany) according to the manufacturer’s instructions and eluted in 30 µL nuclease-free water. Concentration and labeling efficacy of cRNA was determined with a NanoDrop ND-1000 UV-VIS spectrophotometer version 3.2.1 (NanoDrop Technologies, Inc., USA).

### Custom-made Agilent-microarrays for *E*. *gracilis*

Large-scale microarray analysis was performed with the Agilent microarray platform for one-color gene expression analysis. The microarray was designed with the online design tool eArray for SurePrint G3 Custom GE 8 × 60 K microarrays (Design ID 084219, G4102A, Agilent, Santa Clara, USA) on the basis of the new *E*. *gracilis* transcriptome data^2^. Specific 60-mer oligonucleotide probes were designed in sense orientation for 20,396 different transcripts. Slides were printed by Agilent Technologies in a non-contact inkjet printing process with each slide containing eight identical arrays with a total of 62,976 spots. All transcripts under investigation were plotted at least in triplicates with randomized distribution in order to exclude spatial artifacts during hybridization. In addition, 330 *Euglena*-specific replicate probes (control of array reproducibility) as well as 1319 Agilent Technologies controls (spike-in controls, dark and bright corners, and different positive controls) were plotted on the chip.

### Microarray hybridization and wash

Hybridization reactions were performed using the Gene Expression Hybridization Kit (Agilent Technologies, Santa Clara, USA) according to the manufacturer’s instructions for 8-pack microarrays. In brief, 600 ng cRNA was fragmented with 25× fragmentation buffer, mixed with 10× gene expression blocking agent and incubated for 30 min at 60 °C. Subsequently, 2× Hi-RPM hybridization buffer was added to the samples. After centrifugation (13000 rpm, 1 min), samples were loaded onto the array. For every single array on the slide, 40 µL were pipetted into individual gaskets of an Agilent gasket slide and the microarray was placed on top and sealed in a SureHyb hybridization chamber (G2534, Agilent, Santa Clara, USA). The slide was incubated in a rotator rack (G2530-60029, Agilent, USA) in a hybridization oven (G2545A, Agilent Technologies, Santa Clara, USA) for a minimum of 17 h at 65 °C, at a rotational speed of 10 rpm. After overnight incubation, the hybridization chamber was disassembled and the microarray was immediately subjected to stringency washes using the Gene Expression Wash Buffer kit (Agilent, Santa Clara, USA) as recommended by the manufacturer. Slides were washed with wash buffer 1 at room temperature for 1 min, followed by another 1 min wash in wash buffer 2 at approximately 37 °C. Slides were then dried in an oil-free air stream and immediately used for scanning of the microarrays.

### Scanning of the microarrays

Microarray slides were scanned with an Agilent C microarray scanner (G2565CA, Agilent Technologies, Santa Clara, USA). Excitation of cyanin-3-labeled samples was performed with a SHG-YAG laser (emission: 532 nm) in combination with an emission filter (550–610 nm). Fluorescence signals were converted into electrical signals, which were subsequently digitized to 16-bit TIFF files. Settings of the scanner were as follows: Channel Green, resolution 3 µm, scan region Agilent HD 61 × 21.6 mm, TIFF 16-bit, dynamic range 100–10%.

### Evaluation of the microarray scans

The images obtained with the microarray scanner were analyzed with Agilent Feature Extraction software (version 11.5.1.1, Agilent Technologies, Santa Clara, USA). The settings of the extraction protocol were as follows: Extraction protocol: GE1_1105_0ct12, Grid 084219_D_F_20160803, Background method: No Background, Background detrend: On, Multiplicative detrend: True.

### Agilent microarray data analysis and transcript annotation

For normalization, quality assessment and fold change analysis, the microarray data were further processed with GeneSpring 14.8 GX (Agilent Technologies, Santa Clara, USA) software. All samples were normalized by quantile normalization with a baseline set to the median of all samples. Compromised spots as well as outlier samples, as identified by principal component analysis, were removed from further analysis^53^. For hierarchical clustering, ANOVA (one-way) statistical analysis was performed and displayed using the Pearson uncentered (absolute) correlation and Wards linkage rule. The calculation of fold changes was done by the moderated t-test and the p-value computation was performed asymptotically as data were assumed to show normal distribution. Cut-off values for the fold change and p-value were set to 1.5-fold and 0.05, respectively. Each condition was compared to the one before, resulting in the investigation of acceleration-dependent effects: 1 *g* vs 1.8 *g*-1, 1.8 *g*-1 vs µ*g*, µ*g* vs 1.8 *g*-2. At least three independent samples for each acceleration step from different flight days were used for fold change analysis and hierarchical clustering.

For automated transcript annotation, Blast2GO pro (version 4.1.9, BioBam Bioinformatics, Spain) software was used^54–57^, which remotely performs BLAST and InterProScan searches, extracts Gene Ontology (GO) terms as well as enzyme codes (EC)^58–62^. The initial BLAST search was performed based on the protein sequence of the transcript, showing results with an expectation value smaller than 1.0E-3. During mapping and annotation, the BLAST and InterProScan results were linked to their corresponding GO terms from the non-redundant reference protein database (PIR). EC assignment was performed based on GO annotation and visualized via KEGG pathway analysis^63–65^. Furthermore, the gene set enrichment analysis (GSEA) was performed to find regulated functional groups^66^.

## Supplementary information

Figure S1, Tables S1 and S2

## Data Availability

The large-scale data of microarray analysis have been deposited into ArrayExpress. All raw data and detailed experimental protocols can be accessed under the link https://www.ebi.ac.uk/arrayexpress/experiments/E-MTAB-8064.
