# Supplementary material for: Changes of Gene Expression in Euglena gracilis Obtained During the 29th DLR Parabolic Flight Campaign
Source: Sci Rep. 2019 Oct 3;9:14260. doi: 10.1038/s41598-019-50611-4 (PMC6776534; doi:10.1038/s41598-019-50611-4)
Supplement: Supplementary file 1 — Figure S1, Tables S1 and S2 [file 41598_2019_50611_MOESM1_ESM.docx]

**Changes of Gene Expression in *Euglena gracilis* Obtained During the 29^th^ DLR Parabolic Flight Campaign**

Julia Krüger^a^, Peter Richter^a^, Julia Stoltze^a^, Sebastian M. Strauch^a,b^, Marcus Krüger^c^, Viktor Daiker^a^, Binod Prasad^a^, Sophia Sonnewald^d^, Stephen Reid^d^, and Michael Lebert^a*^

^a^ Cell Biology Division: Gravitational Biology Group, Department of Biology, Friedrich-Alexander University Erlangen-Nürnberg, Staudtstraße 5, 91058 Erlangen, Germany

^b^ Postgraduate Program in Health and Environment - University of Joinville Region, Rua Paulo Malschitzki, 10 - Zona Industrial Norte, Joinville - SC - CEP 89219-710, Brazil

^c^ Clinic for Plastic, Aesthetic and Hand Surgery, Otto von Guericke University Magdeburg, Leipziger Straße 44, 39120 Magdeburg, Germany

^d^ Biochemistry Division, Department of Biology, Friedrich-Alexander University Erlangen-Nürnberg, Staudtstraße 5, 91058 Erlangen, Germany

**Supplementary Information**

**Figure S1. Flight hardware for parabolic flight campaigns.** (**A**) Exemplary inside view of the fixation unit showing the PVC sliders, which are manually operated during flight (1) triggering the release of syringes filled with TRIzol^®^ (red). Sterile tubing (3) connects syringes with fixative to the ones filled with *E. gracilis* cells (4). (**B**) Fixation unit in flight configuration mounted inside the aircraft.

**
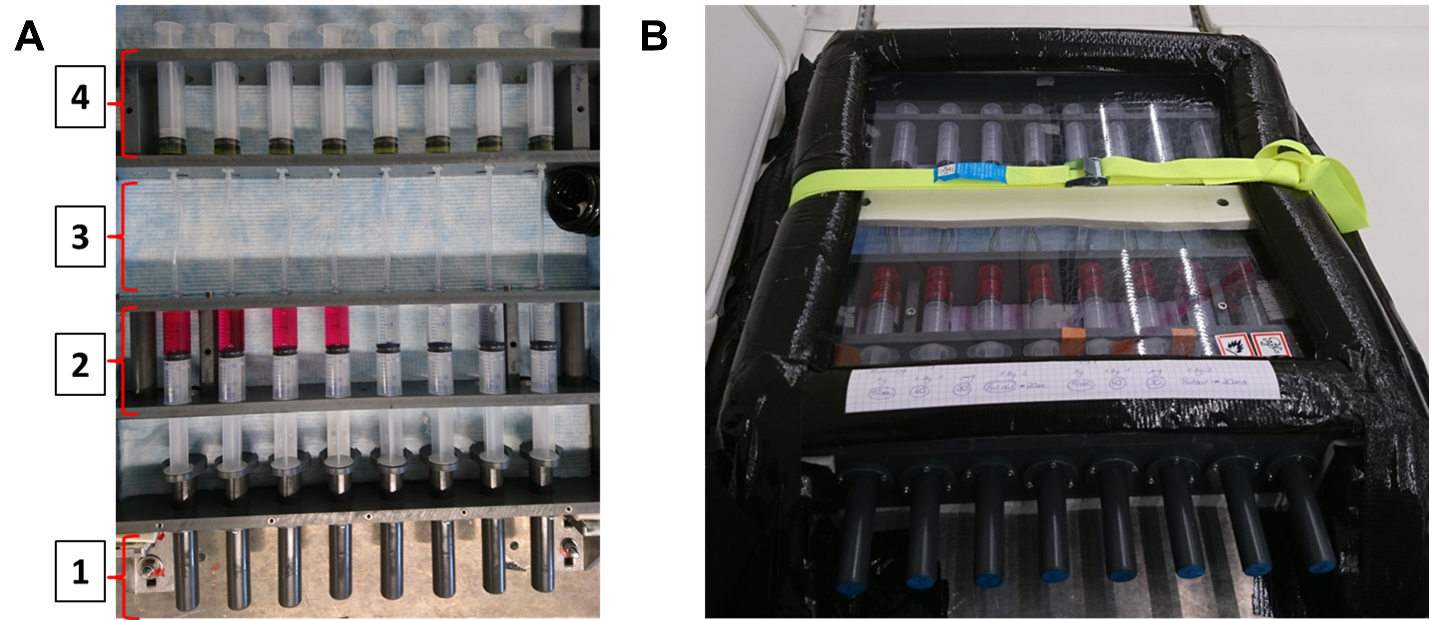
**

Table S1. List of selected transcripts regulated in parabola 1. The specific transcripts (#) are shown with their corresponding sequence description, organism and similarity measure (E-value) found by Blast2GO or manual BLAST search. The fold changes (FC) and p-values of regulation are presented as well.

| **EG_ transcript number #** | **Sequence description** | **Organism** | **E** | **FC** | **p** |
| --- | --- | --- | --- | --- | --- |
| **Parabola 1 1.8 g-1** | | | | | |
| **Signal transduction** | | | | | |
| 15442 | Sensor histidine kinase | *Nocardioides szechwanensis* | 6.90E-04 | 1.96 | 2.70E-03 |
| 13718 | Kinase-like domain-containing | *Kockovaella imperatae* | 6.00E-05 | 1.56 | 3.76E-02 |
| 9153 | Mitogen-activated kinase-binding protein | *Odocoileus virginianus texanus* | 9.00E-86 | -1.61 | 8.91E-04 |
| 3857 | TKL kinase | *Thecamonas trahens* | 4.00E-30 | -1.55 | 3.78E-02 |
| 1113 | Adenylate guanylate cyclase domain-containing protein | *Mycolicibacterium confluentis* | 2.00E-07 | -1.56 | 3.34E-02 |
| 635 | Receptor-type adenylate cyclase GRESAG | *Trypanosoma brucei* | 1.60E-61 | -1.62 | 4.91E-02 |
| 2502 | cAMP specific phosphodiesterase | *Trypanosoma vivax* | 3.10E-65 | 1.59 | 4.14E-03 |
| **Transport** | | | | | |
| 13439 | Calcium-chelator complex transporter | *Drosophila arizonae* | 2.00E-42 | -1.68 | 3.10E-02 |
| 12539 | Phospholipid-transporting ATPase | *Cavenderia fasciculata* | 2.00E-34 | 1.61 | 5.21E-03 |
| 32594 | Sugar transporter SWEET1 isoform X1 | *Mus pahari* | 7.00E-09 | 1.53 | 3.51E-02 |
| **BLUF domain containing protein** | | | | | |
| 31756 | Alpha subunit of photoactivated adenylyl cyclase | *E. gracilis* | 2.90E-41 | -1.72 | 3.78E-02 |
| **Photosynthesis** | | | | | |
| 41739 | Chloroplast light-harvesting complex II precursor Lhcbm3 | *E. gracilis* | 1.20E-15 | -1.52 | 4.57E-03 |
| **Membrane protein** | | | | | |
| 627 | GPI-anchored surface | *Bodo saltans* | 1.80E-109 | -1.98 | 2.53E-02 |
| **Parabola 1 µ*g*** | | | | | |
| **Signal transduction** | | | | | |
| 2195 | Adenylate guanylate cyclase domain-containing protein | *Chrysiogenes arsenatis* | 3.00E-36 | 1.63 | 4.28E-03 |
| 3046 | Receptor-type adenylate cyclase | *Bodo saltans* | 1.30E-31 | 1.5 | 1.18E-02 |
| 9153 | Mitogen-activated kinase-binding protein | *Odocoileus virginianus texanus* | 9.00E-86 | 1.74 | 8.36E-04 |
| **Transport** | | | | | |
| 1269 | Transport protein Sec24-like At3g07100 | *Physcomitrella patens* | 1.00E-165 | -1.54 | 3.12E-02 |
| 2128 | Putative kinesin | *Trypanosoma grayi* | 2.00E-110 | 1.5 | 4.34E-02 |
| 24116 | Cation/H(+) antiporter 19-like protein | *Folsomia candida* | 8.00E-10 | 1.54 | 4.30E-03 |
| 35908 | Potassium transporter | *Magnetospirillum magnetotacticum* | 2.00E-12 | 1.53 | 1.44E-03 |
| 11188 | ABC transporter G family member 31-like | *Gossypium hirsutum* | 2.00E-30 | -1.74 | 2.81E-02 |
| **Membrane protein** | | | | | |
| 10146 | Cell surface protein | *Bacillus cereus* | 2.00E-19 | 1.53 | 8.72E-03 |
| 1870 | Putative transmembrane protein | *Bodo saltans* | 6.90E-32 | 1.57 | 3.74E-02 |
| 627 | GPI-anchored surface | *Bodo saltans* | 1.80E-109 | 1.78 | 4.40E-02 |
| **Parabola 1 1.8 *g*-2** | | | | | |
| **Signal transduction** | | | | | |
| 450 | Mitogen-activated kinase kinase kinase NPK1 | *Oryza sativa* Japonica Group | 9.00E-50 | 1.72 | 4.74E-02 |
| 836 | Hybrid sensor histidine kinase/response regulator | *Opitutus terrae* | 4.90E-37 | 1.52 | 1.27E-02 |
| 8876 | Serine/threonine-protein kinase | *Oryza brachyantha* | 6.00E-48 | -1.63 | 1.61E-02 |
| 21333 | Adenylate guanylate cyclase domain-containing protein | *Bacillus sp.* FJAT-45350 | 6.30E-08 | -1.69 | 2.57E-02 |
| 71885 | Sel1 repeat family protein | *Candidatus Paracaedibacter symbiosus* | 2.70E-10 | -1.53 | 3.15E-02 |
| **Transport** | | | | | |
| 960 | Exportin-4 | *Rhincodon typus* | 7.20E-16 | -1.80 | 7.55E-03 |
| **Photosynthesis** | | | | | |
| 7821 | Carboxyl-terminal-processing peptidase chloroplastic-like isoform X2 | *Physcomitrella patens* | 3.00E-69 | 1.99 | 1.87E-02 |
| **Metabolism** | | | | | |
| 10945 | Carbonic anhydrase | *Oxyrrhis marina* | 7.60E-10 | 1.55 | 5.23E-03 |
| 43521 | Fructose-bisphosphate aldolase 1 | *E. gracilis* | 1.90E-37 | 1.79 | 1.75E-02 |
| **DNA binding** | | | | | |
| 31054 | Myb-like DNA-binding domain-containing | *Neospora caninum Liverpool* | 1.00E-07 | -1.57 | 1.16E-03 |
| **Protein folding** | | | | | |
| 12478 | Tubulin specific chaperone C | *Hondaea fermentalgiana* | 3.00E-24 | 1.68 | 1.66E-02 |
| **Stress protein** | | | | | |
| 33566 | HSP20-like chaperones superfamily protein | *Zea mays* | 3.00E-18 | 1.51 | 1.45E-02 |

Table S2. List of selected transcripts regulated in parabola 31. The specific transcripts (#) are shown with their corresponding sequence description, organism and similarity measure (E-value) found by the Blast2GO or manual BLAST search. The fold changes (FC) and p-values of regulation are presented.

| **EG_ transcript number #** | **Sequence description** | **Organism** | **E** | **FC** | **p** |
| --- | --- | --- | --- | --- | --- |
| **Parabola 31 1.8 *g*-1** | | | | | |
| **Signal transduction** | | | | | |
| 12873 | Kinase domain protein | *Tetrahymena thermophila SB210* | 9.00E-33 | -1.53 | 3.10E-02 |
| 14876 | Serine/threonine-protein phosphatase 6 regulatory ankyrin repeat subunit B-like | *Strongylocentrotus purpuratus* | 2.00E-30 | -1.60 | 1.03E-03 |
| **Transport** | | | | | |
| 590 | Phospholipid-transporting ATPase 9 | *Ipomoea nil* | 0.00E+00 | -1.51 | 1.72E-02 |
| 35058 | BMP family ABC transporter substrate-binding protein | *Lachnoclostridium sp. An298* | 2.60E-11 | 2.03 | 3.87E-03 |
| **Membrane** | | | | | |
| 13677 | Hypothetical protein | *Bathycoccus prasinos* | 2.30E-11 | 1.59 | 2.37E-02 |
| 2251 | Transmembrane protein, putative | *Bodo saltans* | 5.30E-43 | -1.60 | 2.36E-02 |
| **Protein modification** | | | | | |
| 17721 | E3 ubiquitin- ligase Midline-1-like isoform X1 | *Callorhinchus milii* | 1.20E-14 | 1.51 | 3.47E-02 |
| 38952 | E3 ubiquitin- ligase TRIM39-like | *Oncorhynchus mykiss* | 4.90E-10 | -1.60 | 1.15E-02 |
| 59672 | Ubiquitin carboxyl-terminal hydrolase | *Phoenicopterus ruber ruber* | 6.40E-14 | 1.54 | 3.95E-02 |
| 809 | Ubiquitin carboxyl-terminal hydrolase 5 | *Dacryopinax primogenitus* | 4.00E-97 | 1.72 | 4.98E-02 |
| **Stress protein** | | | | | |
| 14035 | Dual specificity protein phosphatase 10 isoform X4 | *Cimex lectularius* | 7.00E-28 | -1.69 | 3.93E-02 |
| **Parabola 31 µ*g*** | | | | | |
| **Signal transduction** | | | | | |
| 18176 | Cyclin-dependent kinase-like 2 | *Chrysochromulina sp.* CCMP291 | 3.60E-140 | -1.82 | 3.07E-03 |
| 27577 | Adenylate guanylate cyclase domain-containing protein | *Candidatus Koribacter versatilis* | 2.30E-13 | -1.62 | 5.37E-03 |
| 3122 | Cyclic AMP-dependent protein kinase-like protein regulatory subunit | *Pythium insidiosum* | 1.70E-20 | 1.51 | 3.11E-02 |
| 31490 | Ankyrin-1-like protein | *Crassostrea gigas* | 5.00E-18 | -1.89 | 3.19E-02 |
| 37201 | Receptor-type adenylate cyclase | *Bodo saltans* | 9.50E-05 | 1.55 | 4.09E-03 |
| 450 | Mitogen-activated kinase kinase kinase NPK1 | *Oryza sativa Japonica Group* | 9.00E-50 | -1.57 | 1.39E-02 |
| 5221 | PAS domain-containing sensor histidine kinase | *Skermanella aerolata* | 3.00E-27 | 1.51 | 2.69E-02 |
| 9749 | Adenylate/guanylate cyclase domain-containing protein | *Legionella cincinnatiensis* | 1.00E-54 | 1.50 | 1.95E-02 |
| **Transport** | | | | | |
| 39876 | Sugar nucleotid transporter | *Chlamydomonas reinhardtii* | 2.00E-21 | 1.59 | 2.39E-02 |
| 4425 | ATP-binding cassette sub-family A member 1 | *Aurantiochytrium sp.* FCC1311 | 1.30E-52 | -1.65 | 3.76E-02 |
| **Membrane** | | | | | |
| 32155 | DUF4954 domain-containing | *Candidatus Omnitrophica bacterium* | 3.40E-27 | 1.75 | 2.99E-02 |
| **Calcium-binding proteins** | | | | | |
| 7280 | Phosphatidylinositol-4-phosphate 5-kinase | *Ichthyophthirius multifiliis* | 4.70E-31 | -1.54 | 2.27E-02 |
| 7955 | C2 domain protein | *-* | - | -1.62 | 1.88E-02 |
| **Motor protein** | | | | | |
| 23041 | Kinesin | *Bodo saltans* | 5.70E-17 | -1.54 | 2.27E-02 |
| **Stress protein** | | | | | |
| 1003 | Peroxisomal membrane protein mpv17 | *Chrysochromulina sp.* CCMP291 | 9.00E-44 | 1.50 | 3.11E-02 |
| **Energy production and conversion** | | | | | |
| 8836 | NADP malic enzyme | *Chlorella sorokiniana* | 1.60E-172 | -1.53 | 2.11E-02 |
| **Parabola 31 1.8 *g*-2** | | | | | |
| **Signal transduction** | | | | | |
| 1113 | Adenylate guanylate cyclase domain-containing protein | *Mycobacterium confluentis* | 9.20E-07 | 1.70 | 9.19E-03 |
| 13761 | Kinase domain protein | *Tetrahymena thermophila* SB210 | 1.40E-21 | -1.76 | 9.53E-03 |
| 14406 | Receptor-type adenylate cyclase | *Trypanosoma vivax* Y486 | 1.20E-07 | 1.59 | 3.20E-02 |
| 18176 | Cyclin-dependent kinase-like 2 | *Chrysochromulina sp. CCMP291* | 3.60E-140 | 1.72 | 3.98E-03 |
| 2011 | Adenylate guanylate cyclase domain-containing | *Bodo saltans* | 1.80E-09 | -1.52 | 1.29E-02 |
| 21102 | Arrestin domain-containing protein 2 | *Tetrabaena socialis* | 5.00E-11 | -1.53 | 7.97E-04 |
| 22840 | Ubiquitin thioesterase trabid-like | *Atta colombica* | 3.30E-29 | -1.96 | 6.79E-03 |
| 22951 | Magnesium-dependent phosphatase 1 | *Eurytemora affinis* | 2.10E-34 | 1.53 | 3.29E-02 |
| 27577 | Adenylate guanylate cyclase domain-containing protein | *Candidatus Koribacter versatilis* | 2.30E-13 | 1.75 | 4.06E-03 |
| 38932 | Adenylate cyclase type 3-like | *Oncorhynchus mykiss* | 1.70E-07 | 1.58 | 2.66E-02 |
| 8602 | TKL/IRAK protein kinase | *Capsaspora owczarzaki ATCC 30864* | 1.00E-51 | 1.71 | 8.23E-03 |
| **Transport** | | | | | |
| 1810 | Ammonium transporter | *Micractinium conductrix* | 5.00E-57 | 1.64 | 2.12E-03 |
| 8617 | ABC transporter A family protein | *Oxytricha trifallax* | 3.00E-17 | 1.67 | 5.87E-05 |
| **Membrane** | | | | | |
| 15691 | Related to Slh1p interacting factor | *Serendipita indica* | 3.00E-31 | 2.07 | 1.79E-02 |
| 2724 | Multiple C2 and transmembrane domain-containing protein 1 | *Trichinella pseudospiralis* | 3.70E-04 | 1.82 | 4.07E-02 |
| 627 | GPI-anchored surface | *Bodo saltans* | 1.80E-109 | -1.95 | 4.42E-02 |
| **Actin-binding protein** | | | | | |
| 22888 | Actin binding protein | *Entamoeba dispar SAW760* | 5.90E-22 | -1.56 | 3.98E-02 |
| **Microtubuli-binding protein** | | | | | |
| 38089 | Centrosomal protein of 164 kDa | *Nestor notabilis* | 1.00E-31 | 1.75 | 3.09E-02 |
| **Cytoskeleton** | | | | | |
| 800 | Trichohyalin | *Trypanosoma grayi* | 3.00E-33 | -1.53 | 1.57E-02 |
| **Motor protein** | | | | | |
| 14051 | Dynamin-related protein | *Ectocarpus siliculosus* | 5.00E-67 | -1.52 | 9.17E-03 |
| **Protein modification** | | | | | |
| 3282 | Ubiquitin-conjugating enzyme E2 Z-like | *Planoprotostelium fungivorum* | 1.30E-47 | -2.00 | 3.26E-02 |
| 36027 | E3 ubiquitin- ligase Midline-1 isoform X1 | *Branchiostoma belcheri* | 7.20E-21 | 1.60 | 4.61E-02 |
| 5320 | E3 ubiquitin- ligase | *Plasmopara halstedii* | 2.00E-28 | -1.86 | 3.16E-02 |
| 5328 | E3 ubiquitin-protein ligase TRIM39-like isoform X2 | *Callorhinchus milii* | 2.00E-04 | 1.68 | 3.38E-02 |
| 5448 | NHL repeat containing protein | *Chrysochromulina sp. CCMP291* | 3.60E-59 | 1.64 | 1.39E-02 |
| **Energy production and conversion** | | | | | |
| 8836 | NADP malic enzyme | *Chlorella sorokiniana* | 1.60E-172 | 1.67 | 3.59E-02 |
| **Chromatin organization** | | | | | |
| 26357 | Reverse transcriptase domain protein | *Umbilicaria pustulata* | 6.00E-11 | -1.65 | 1.88E-02 |
